# No observed effect on brain vasculature of Alzheimer’s disease-related mutations in the zebrafish presenilin 1 gene

**DOI:** 10.1186/s13041-021-00734-5

**Published:** 2021-01-25

**Authors:** Karissa Barthelson, Morgan Newman, Cameron J. Nowell, Michael Lardelli

**Affiliations:** 1 Alzheimer’s Disease Genetics Laboratory, School of Biological Sciences, University of Adelaide, North Terrace, Adelaide, SA 5005 Australia; 2 Drug Discovery Biology, Monash Institute of Pharmaceutical Sciences, Monash University, Parkville, VIC 3058 Australia

**Keywords:** Zebrafish, Vasculature, Confocal laser scanning microscopy, 3D reconstruction

## Abstract

Previously, we found that brains of adult zebrafish heterozygous for Alzheimer’s disease-related mutations in their presenilin 1 gene (*psen1,* orthologous to human *PSEN1*) show greater basal expression levels of hypoxia responsive genes relative to their wild type siblings under normoxia, suggesting hypoxic stress. In this study, we investigated whether this might be due to changes in brain vasculature. We generated and compared 3D reconstructions of GFP-labelled blood vessels of the zebrafish forebrain from heterozygous *psen1* mutant zebrafish and their wild type siblings. We observed no statistically significant differences in vessel density, surface area, overall mean diameter, overall straightness, or total vessel length normalised to the volume of the telencephalon. Our findings do not support that changes in vascular morphology are responsible for the increased basal expression of hypoxia responsive genes in *psen1* heterozygous mutant brains.

## Introduction

The dominant hypothesis of Alzheimer’s disease (AD) pathogenesis is the amyloid cascade hypothesis (ACH) [1], which postulates the amyloid β peptide (Aβ) as initiating a pathological process resulting in neurodegeneration and dementia (reviewed in (2)). An alternative to the ACH is the vascular hypothesis [3], asserting that age-related cerebral vascular abnormalities induce AD pathologies by limiting nutrient and oxygen delivery to produce hypoxic stress, a neural energy crisis and, consequently, neurodegeneration. Significant evidence supports the vascular hypothesis of AD (reviewed in [4]).

Rare, inherited forms of AD are caused by dominant mutations in a small number of genes (early-onset familial AD, EOfAD). Most EOfAD cases are due to heterozygous mutations in the gene presenilin 1 (*PSEN1*) that obey a “reading-frame preservation rule” [5]. Mutations allowing production of a transcript(s) with an altered coding sequence but, nevertheless, utilising the original stop codon cause EOfAD while mutant alleles coding only for truncated proteins do not. We previously generated knock-in models in zebrafish with each of these types of mutant *psen1* allele: K97Gfs, a frameshift mutation encoding a truncated protein similar to the human PS2V isoform that is increased in sporadic, late onset AD [6], and Q96_K97del: an EOfAD-like, reading-frame-preserving deletion of two codons [7].

We recently observed in normoxic adult zebrafish brains that heterozygosity for either of the above two mutations causes increased basal expression levels of hypoxia responsive genes (HRGs, genes with expression regulated by a master regulator of the transcriptional response to hypoxia: hypoxia-inducible factor 1 (HIF1)). This implied that the heterozygous *psen1* mutant fish brains were already under some form of hypoxic stress [8], possibly due to changes in vasculature, as have been observed in transgenic mice expressing human *PSEN1* EOfAD mutation-bearing transgenes in neurons [9]. Therefore, we examined the effects on forebrain vasculature with age of heterozygosity for the K97Gfs and Q96_K97del mutations of *psen1* by exploiting the *fli1:GFP* transgene that labels zebrafish endothelial cells [10].

## Methods

**Fig. 1:**
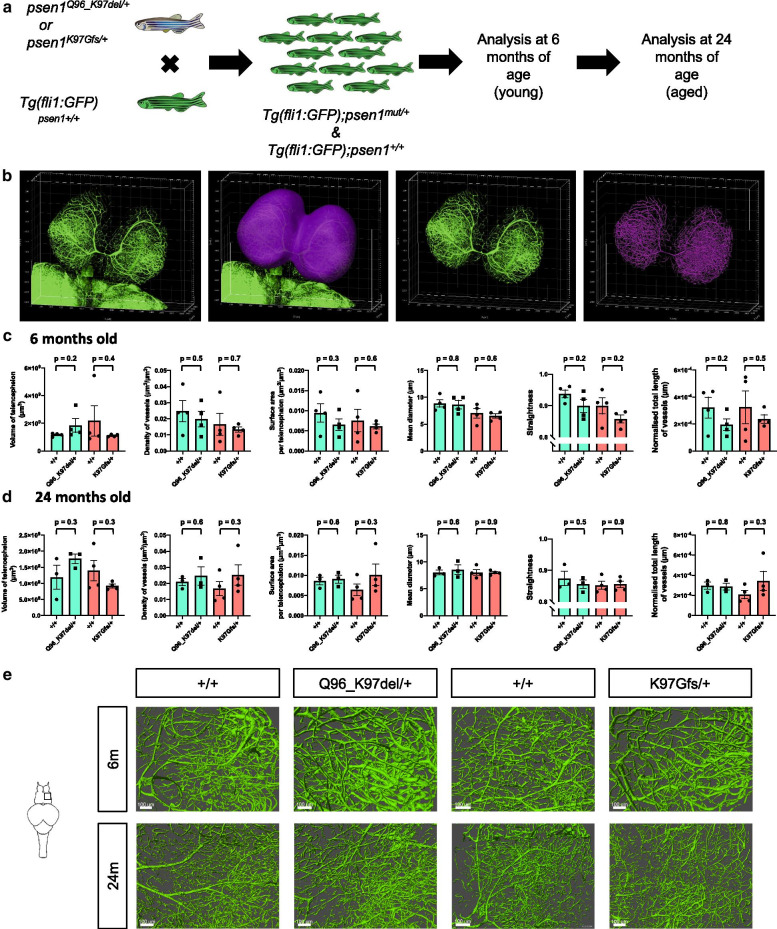
No statistically significant changes to brain vascular network parameters due to heterozygosity for the Q96_K97del or K97Gfs mutations of *psen1. a* Experimental design flow diagram. Genome-edited *psen1* heterozygous mutant fish were pair-mated with transgenic zebrafish expressing green fluorescent protein (GFP) under the control of the *fli1* promotor (*fli1:GFP* transgene). GFP-fluorescent larvae were selected to give a family of transgenic siblings either wild type or heterozygous for a *psen1* mutation. Analysis of the brain vascular network was performed at 6 and 24 months of age. **b** 3D image analysis pipeline. The telencephalon was manually segmented from the optic tectum using contour lines to generate a masked surface channel containing only GFP signals from the telencephalon. Then, an additional surface was generated over the vessels to remove background fluorescence. A masked surface channel was generated from this surface as input for the filament trace algorithm. **c** Measured values from the surface and filament trace algorithms for the 6 month old zebrafish and **d** the 24 month old female zebrafish for (left to right) the volume of the telencephalon, the density of *fli1:GFP* positive vessels per telencephalon, the surface area of vessels normalised to the volume of the telencephalon, the overall mean diameter of vessels, the overall straightness of the vessels, and the total length of the vessels normalised to the volume of the telencephalon. Data are presented as the mean ± standard deviation. Colours of the bars represent the two families of fish used in this analysis. *P*-values were determined by Student’s *t*-test assuming unequal variance. **e** Representative images of a 200 µm section of the right hemisphere of the telencephalon from fish of each age and genotype. Scale bars indicate 100 µm. Vessels appeared morphologically similar in each age and genotype

Single zebrafish heterozygous for either *psen1* mutation were mated with single fish bearing the *fli1::GFP* [10] transgene. GFP-fluorescent progeny were selected to form families of siblings either wild type or heterozygous for the *psen1* mutant alleles (Fig. 1a). We used n = 4 brains of each sibling genotype at 6 months (young adult) and 24 months (aged) of age for tissue clearing using the PACT method [11]. Briefly, PACT involves infusing and crosslinking the brain with an acrylamide-based hydrogel. Then, light scattering lipids are passively removed by incubating the brain with a detergent, allowing light to penetrate deep into the tissue [11, 12]. We imaged the telencephalons (thought to be the region loosely equivalent of the prefrontal cortex in humans) using an Olympus FV3000 confocal microscope, and performed 3D image analysis using Imaris v9.1 (Bitplane) (Fig. 1b). For a detailed description of methods, see Additional File 1.

## Results and conclusion

No statistically significant differences between sibling genotypes at each age were observed for any of the measured parameters (see Fig. 1). This does not support that the increased basal levels of HRGs observed previously in our zebrafish *psen1* mutants are due to vascular changes. However, subtle changes to vasculature due to *psen1* genotype may be too small to detect using this method and further experimentation using a larger number of biological replicates may increase statistical power to detect changes to these measured parameters. Alternatively, other factors such as altered γ-secretase activity [13] and/or cellular ferrous iron levels [14] may influence HIF1-⍺ activity to affect basal HRG expression.

## Supplementary Information

**Additional file 1.** Detailed description of sample preparation, imaging and 3D image analysis. 

**Additional file 2.** Quantified values used to produce the graphs in Fig. 1.

## Data Availability

The quantified values used to produce the graphs in Fig. 1 can be found in Additional File 2. Raw microscopy images from the current study are available from the corresponding author upon reasonable request.

## Abbreviations

ACH: Amyloid cascade hypothesis
AD: Alzheimer’s disease
Aβ: Amyloid beta
EOfAD: Early-onset familial Alzheimer’s disease
HIF1: Hypoxia-inducible factor 1
HIF1-⍺: Hypoxia-inducible factor 1, alpha subunit
HRG: Hypoxia response gene
PSEN1: Presenilin 1
