# Supplementary material for: No observed effect on brain vasculature of Alzheimer’s disease-related mutations in the zebrafish presenilin 1 gene
Source: Mol Brain. 2021 Jan 25;14:22. doi: 10.1186/s13041-021-00734-5 (PMC7831246; doi:10.1186/s13041-021-00734-5)
Supplement: Supplementary file 1 — Additional file 1. Detailed description of sample preparation, imaging and 3D image analysis. [file 13041_2021_734_MOESM1_ESM.docx]

# Additional File 1: Detailed Methods

Two families of zebrafish were generated comprised of sibling fish either wild type for *psen1* or heterozygous for either the K97Gfs or Q96_K97del *psen1* mutation and also carrying a copy of the *fli1:GFP* transgene as described in the main text. Each family of siblings was raised together in the same tank, to reduce environmental variation between individuals. To reduce further any variation due to sex, we chose to analyse only female zebrafish, as AD incidence is higher in human females. At 6 months of age, we sacrificed 12 females of a tank and removed their entire heads for PACT clearing, as well as a small piece of each fish’s tail for genotyping by polymerase chain reactions (PCRs). The fish remaining in a tank were allowed to develop to 24 months of age, at which time the head processing and genotyping was repeated.

### PCR genotyping

Genomic DNA was extracted from the fin biopsies by incubating for 3 hours at 55 °C in Proteinase K solution (Roche, Basel, Switzerland) diluted to a working concentration of 1 mg/mL in 1 x TE buffer. To inactivate proteinase K, the genomic DNA preparations were incubated at 95 °C for 5 minutes. Then, debris was sedimented by centrifugation with a relative centrifugal force of 16,100 for 3 minutes. The supernatants containing genomic DNA were then transferred to clean tubes for subsequent allele-specific PCRs.

Allele-specific PCRs were performed using GoTaq DNA polymerase (Promega, Madison, Wisconsin, USA) and primers specific to each of the *psen1* alleles present in the fish (see **Table 1**). Of the 12 female fish sacrificed, n = 4 female fish of each genotype were used for PACT clearing and imaging (except for the 24 month old Q96_K97del group, where only 3 female fish of each genotype remained in the tank). The observer performing the image analysis was blinded to genotype until after the analysis was complete.

| **Table 1: Genotyping primer sequences** | |
| --- | --- |
| **Primer name** | **Sequence (5’ to 3’)** |
| Q96_K97del F | TGTCAGCTTCTACACAGACGGA |
| K97Gfs F | AATCTGTCAGCTTCTACACACAAGG |
| psen1 WT F | TCTGTCAGCTTCTACACACAGAAGG |
| Common R | CCATCCCTAAACTGCTCCTACTC |

### PACT tissue clearing

To perform PACT tissue clearing, the entire heads from the 4 female fish per genotype at each age were fixed in 8 mLs of 4% paraformaldehyde (PFA) overnight at 4°C with gentle rocking. The brains were then carefully removed from the skull using watchmakers’ forceps in 1 x phosphate-buffered saline (PBS). Once the brains were removed, they were protected from light to prevent any bleaching of the endogenous fluorophores. The fixed brains were then placed in 10 mLs of cold hydrogel solution (0.25% VA-044, 1% acrylamide/bis acrylamide, 4% PFA in 1 x PBS) and incubated at 4 °C for 3 days with gentle rocking to infuse the brain with the hydrogel monomers. To polymerise the hydrogel, we first minimised the oxygen levels in the tubes by placing them in a hypoxia chamber with an Anaerogen^TM^ 2.5L sachet for 2.5 hours, followed by incubation of the entire sealed hypoxia chamber at 37°C for 3 hours. To remove lipids from the brain, we incubated the brains in 10 mL of clearing solution (4% SDS, 200mM boric acid, pH 8.5) for 7 days at 37 °C with gentle rocking and replacing the clearing solution with fresh solution each day. We then washed away the clearing solution with several washes of 1 x PBST (0.1% Triton-X100 in 1 x PBS) and brains were stored at room temperature protected from light until imaging.

### Confocal laser scanning microscopy image acquisition

For confocal laser scanning microscopy (CLSM), the cleared brains were refractive index-matched using a homemade refractive index matching solution (sRIMS, (2)). We then mounted the brains on a CoverWell™ Imaging Chamber Gasket (1mm depth, Invitrogen, Carlsbad, CA, USA), filled with sRIMS with the dorsal side of the brain facing the coverslip. We then imaged the telencephalon using an Olympus FV3000 confocal microscope using the 10X2 UPLSAPO objective (dry, numerical aperture = 0.4, working distance = 3.1 mm) and Olympus Fluoview software. A 488 nm laser was used to illuminate the sample, and the emission spectrum captured was light of wavelengths between 500 nm and 600 nm. The scanning was performed at 2µs/pixel with a size of 1024 x 1024 pixels. The laser power (%), HV (sensitivity) and offset were optimised for each brain and line averaging was set to 3. Z-stacks were taken from the dorsal to the ventral extent of the brain, with each image in the stack spaced 3.61 µm apart. The Bright Z function of Olympus Fluoview software was used to increase the laser power and sensitivity with increasing z. A multi area time lapse (MATL) was used to capture the entire telencephalon (time series turned off). Then the resulting z-stacks were stitched together using the algorithm in the Olympus Fluoview software package. Imaging took approximately 9 hours per brain, and generated a final file size of approximately 13 GB.

### 3D image analysis

3D reconstruction of brain vascular networks was performed using Imaris (BitPlane). For each image, we manually segmented out the telencephalon from the optic tectum and the olfactory bulbs using contour lines in the surface tool. We then used the automated tool for surface generation to generate a surface over the GFP-positive vessels in the telencephalon. This surface was used to create a masked surface channel which removed background fluorescence. We then performed a filament trace using the “Threshold (loops)” algorithm using the default parameters on the masked surface channel to generate volume, surface area, total length, mean diameter and straightness values for each brain. Pairwise comparisons between the *psen1* mutant fish with their wild type siblings for each of these parameters was performed using Student’s *t*-test assuming unequal variance.

### References

1. Chung K, Wallace J, Kim S-Y, Kalyanasundaram S, Andalman AS, Davidson TJ, et al. Structural and molecular interrogation of intact biological systems. Nature. 2013;497:332.

2. Yang B, Treweek Jennifer B, Kulkarni Rajan P, Deverman Benjamin E, Chen C-K, Lubeck E, et al. Single-Cell Phenotyping within Transparent Intact Tissue through Whole-Body Clearing. Cell. 2014;158(4):945-58.
